# The Application of a Novel Ceramic Liner Improves Bonding between Zirconia and Veneering Porcelain

**DOI:** 10.3390/ma10091023

**Published:** 2017-09-02

**Authors:** Hee-Sung Lee, Tae-Yub Kwon

**Affiliations:** 1 Department of Dental Science, Graduate School, Kyungpook National University, 2-188-1 Samduk-dong, Jung-gu, Daegu 700-412, Korea; yhs6770@knu.ac.kr; 2 Department of Dental Biomaterials, School of Dentistry and Institute for Biomaterials Research & Development, Kyungpook National University, 2-188-1 Samduk-dong, Jung-gu, Daegu 700-412, Korea

**Keywords:** zirconia, veneering porcelain, bonding, ceramic liner

## Abstract

The adhesion of porcelain to zirconia is a key factor in the success of bilayered restorations. In this study, the efficacy of a novel experimental liner (EL) containing zirconia for improved bonding between zirconia and veneering porcelain was tested. Four ELs containing various concentrations (0, 3.0, 6.0, and 9.0 wt %) of zirconia were prepared. Testing determined the most effective EL (EL3 containing 3.0 wt % zirconia) in terms of shear bond strength value (*n* = 15). Three different bar-shaped zirconia/porcelain bilayer specimens were prepared for a three-point flexural strength (TPFS) test (*n* = 15): no-liner (NL), commercial liner (CL), and EL3. Specimens were tested for TPFS with the porcelain under tension and the maximum load was measured at the first sign of fracture. The strength data were analyzed using one-way ANOVA and Tukey’s test (*α* = 0.05) as well as Weibull distribution. When compared to NL, the CL application had no effect, while the EL3 application had a significant positive effect (*p* < 0.001) on the flexural strength. Weibull analysis also revealed the highest shape and scale parameters for group EL3. Within the limitations of this study, the novel ceramic liner containing 3.0 wt % zirconia (EL3) significantly enhanced the zirconia/porcelain interfacial bonding.

## 1. Introduction

When highly esthetic restorations are required in clinical situations, all-ceramic restorations are often preferred over metal-ceramic restorations [1,2]. Pure zirconia has a monoclinic phase at room temperature, but the addition of 3 mol% yttria creates yttria-stabilized tetragonal zirconia polycrystal (Y-TZP) [3]. Retention of the tetragonal phase at room temperature provides mechanical properties superior to other all-ceramic systems due to the tetragonal-to-monoclinic phase transformation [4,5,6,7]. Thus, Y-TZP has drawn attention recently as a core material for all-ceramic restorations [7].

Although zirconia (Y-TZP) materials are more translucent than metal alloys [8,9], they have the considerable drawback of being essentially white and opaque [10]. Thus, zirconia-based restorations are usually covered by a (more translucent) veneering ceramic for enhanced esthetic quality. However, these two ceramics have significant differences in their chemistry and microstructures and, consequently, in certain mechanical properties [9]. Although resin bonding to zirconia has become a topic of great interest in recent years [2,8], another specific problematic area is the nature of the interface between the veneering porcelain and the zirconia substructure [11]. The mechanical integrity and adhesion of the porcelain to the zirconia framework are key factors in the success of veneer/core bilayered restorations [7]. It has been reported that the incidence of veneering porcelain fracture in zirconia-based restorations is relatively high when compared with porcelain-fused-to-metal restorative systems [2,12]. Delamination (failure at the porcelain/zirconia interface) and chipping (failure within the veneering porcelain) are the two most common failure modes [2,13]. Several potential explanations for such fracture behavior have been reported, including: the processing technique of the veneering porcelain, its thickness, as well as the cooling protocol [6,14]. In particular, it has been reported that the cooling rate, core-veneer thickness ratio, and mismatch of the coefficient of linear thermal expansion (CTE) are important factors in the development of residual stresses [15,16], which greatly influence the strength and fracture characteristics of zirconia-based restorations [17].

Failures in zirconia-based restorations either originate from the veneer and propagate to the interface or originate from the zirconia/veneer interface [6,18]. Voids and flaws inevitably exist at the interface, and crack may initiate from these voids and flaws due to stress concentration under a certain loading [18]. Various surface conditioning methods of zirconia substructure have been recommended for enhanced zirconia/porcelain bonding. Among them, air-abrasion and ceramic liner application have been the most researched [6,12,19,20]. Air-abrasion of zirconia with alumina particles may increase the surface roughness and provide undercuts for bonding with veneering porcelain [12]. A ceramic liner material may also be used prior to veneering to mask the bright white appearance of zirconia and improve the wetting [2,10,12]. However, the effects of the air-abrasion and the liner application on the adhesion between the zirconia substructure and veneering porcelain remain controversial. Kim et al. [12] suggested that the air-abrasion of zirconia was useful for increasing the interfacial bond strength, but that application of a liner increased the possibility of failure at the interface. Wang et al. [19] also demonstrated that liner application prior to veneering reduces the interfacial bond strength. On the contrary, a few studies showed that the liner application enhanced the bond strength between some veneering porcelains and a zirconia framework [21,22]. Benetti et al. [6] reported that the use of a liner had no effect on the flexural strength nor the failure mode of the ceramic bilayer system tested. Wang et al. [18] suggested that air-abrasion and liner application reduced zirconia/veneer interfacial toughness.

The chemical composition of commercial ceramic liners varies depending on the manufacturer, but the primary component is silica (SiO_2_), indicating a similar composition to veneering porcelain [12,19]. Thus, the mechanical properties of a ceramic liner resemble those of veneering porcelains and a stronger bond is expected at the porcelain/liner interface than at the liner/zirconia interface [19]. Hence, it was assumed that a ceramic liner containing zirconia would improve its mechanical properties and adhesion to the liner/zirconia interface as well as to the porcelain/liner interface.

Therefore, the purpose of this in vitro study was to evaluate the efficacy of the application of a novel zirconia-containing ceramic liner on the bonding between zirconia and veneering porcelain, using shear bond strength (SBS) and three-point flexural strength (TPFS) tests, and to compare it to those of a no-liner application and a commercial ceramic liner application. Table 1 summarizes the brand names, manufacturers, chemical compositions, and batch numbers of the zirconia, veneering ceramic, and commercial liner (CL) used in this study. In addition, four glycerol-based experimental liners (ELs) containing 0, 3.0, 6.0, and 9.0 wt % zirconia powders with a particle size of 90 nm were prepared (code: EL0, EL3, EL6, and EL9, respectively), as also detailed in Table 1. The hypothesis of this study was that the application of a novel ceramic liner to a zirconia substructure prior to veneering would not improve bonding between zirconia and porcelain.

## 2. Results

### 2.1. Shear Bond Strength (SBS) Test

Table 2 represents the SBS test results together with the Weibull distribution values. Group EL3 achieved the highest value among all the groups tested, the difference being statistically significant (*p* < 0.001). None of the other groups showed any statistically significant differences (*p* > 0.05). However, group CL exhibited a lower Weibull modulus (*m* = 4.7) than group NL (*m* = 7.6). Group EL3 showed the highest Weibull modulus (*m* = 8.9) among all the groups tested. Most of the fractured specimens showed a combination of cohesive and adhesive failure modes, in which residual porcelain layer remained on the zirconia surfaces. Group CL produced adhesive failure in three specimens (*n* = 3/15).

### 2.2. Coefficient of Linear Thermal Expansion (CTE)

The CTE values of the four ceramic materials tested are shown in Table 3. The veneering porcelain showed a slightly lower CTE value (9.1 ± 0.1) than the zirconia material (10.7 ± 0.1). The values for groups CL and EL3 (9.6 ± 0.1 and 9.5 ± 0.2, respectively) were positioned between the zirconia and the veneering porcelain, as there was no significant difference in the values between the two liners (*p* = 0.290).

### 2.3. Three-Point Flexural Strength (TPFS) Test

**Figure 1:**
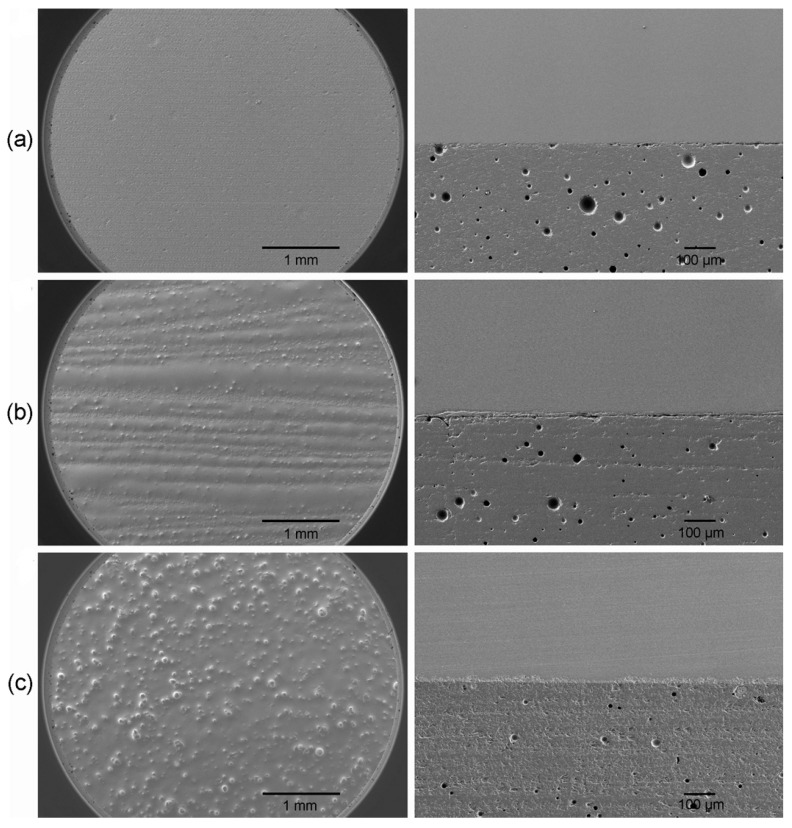
SEM image showing the zirconia specimen surface for each group (**Left**, 25×, **b**,**c**: taken after firing of the liners) and the cross-sectional morphology of zirconia/porcelain interface (**Right**, 100×). (**a**): Group NL; (**b**): Group CL; and (**c**): Group EL3.

Figure 1 shows the SEM images of the zirconia specimen surfaces for groups NL, CL, and EL3 as well as cross-sectional morphologies of the zirconia/porcelain interfaces. Group EL3 showed a more homogeneously dispersed liner on the zirconia surface than group CL. In addition, more intimate contact along the interface between zirconia and veneering porcelain was observed in group EL3, whereas greater gap formations at the interface were detected in groups NL and CL.

**Figure 2:**
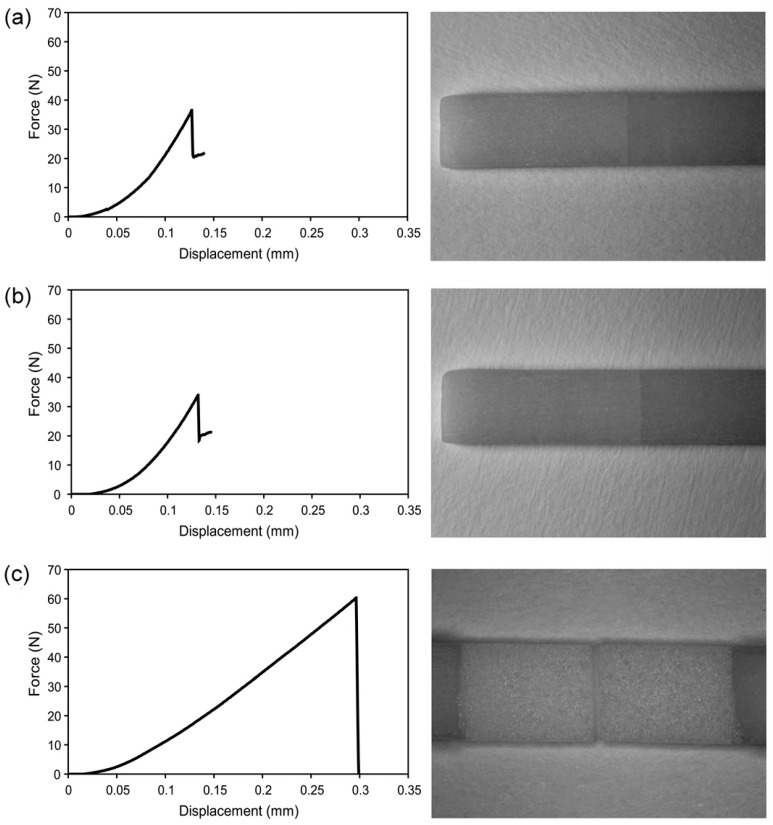
Force-displacement graphs during three-point flexural strength test (**Left**) and optical images of the corresponding fractured specimens (**Right**; 8× (**a**,**b**) and 10× (**c**)). (**a**): A specimen of group NL showed cracking; (**b**): A specimen of group CL showed cracking; and (**c**): A specimen of group EL3 showed catastrophic failure. See Table 4 for detailed failure mode results.

Figure 2 and Table 4 show the TPFS test results for the three groups. The Weibull distribution values are also given in Table 4. Group EL3 showed a significantly higher flexural strength value (*p* < 0.001) than the other two groups, which showed statistically similar values (*p* = 0.618). Group EL3 also exhibited the highest Weibull modulus (*m* = 7.3), followed by group NL (*m* = 6.0) and then group CL (*m* = 2.7). For groups NL and CL, most of the fractured specimens showed cracking of the veneering porcelain reaching the porcelain/zirconia interface. On the contrary, approximately one-third (*n* = 6/15) of the group EL3 specimens underwent catastrophic failure involving both the veneering porcelain and zirconia. In group EL3, the partially fractured zirconia surface was covered by either the liner or porcelain layer.

## 3. Discussion

In this in vitro study, novel glycerol-based ELs containing various concentrations of zirconia were prepared and their efficacy for improved bonding between zirconia and veneering porcelain was tested. In a preliminary test, ELs containing zirconia powders were not sintered at the firing temperature of the CL. Therefore, cryolite (Na_3_AlF_6_), which is a strong fluxing agent, was additionally incorporated into the ELs containing zirconia powders in order to control the firing temperature (Table 1). In this in vitro study, the EL3 application had a significant positive effect on the TPFS of the bilayer ceramic specimens as well as on the SBS of the bonded specimens (Table 2 and Table 4). Moreover, Weibull analysis revealed the highest shape and scale parameters for group EL3 in both tests. Therefore, the hypothesis that that the application of an EL to zirconia substructure prior to veneering would not improve the bonding has to be rejected.

**Figure 3:**
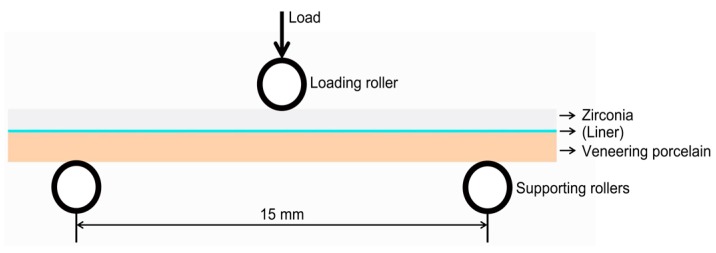
Three-point flexural strength test specimen and test system in a universal testing machine.

To evaluate bonding between a veneering ceramic and a zirconia framework, different test methods, including bond strength tests and flexural tests, have been suggested [2,10]. Although the SBS test is common in the literature [7,10,12,23], it has disadvantages such as the development of non-homogenous stress distribution in the bonding surface and a dependency on the elastic modulus of bonding [2,10]. In the TPFS test, maximum stresses are created at the porcelain surface, resulting in predictable tensile failures (Figure 3) [2,10,24]. However, an adequate standardized test setup and a minimum required bond strength have still not been established [7]. In this study, the two different test methods were used together to evaluate the bonding between veneering porcelain and zirconia. In addition, the SBS and TPFS test results were analyzed in terms of Weibull distribution parameters. Structural reliability of dental ceramics is a major factor in the clinical success of ceramic restorations [25]. The Weibull modulus and the Weibull characteristic strength provide a more accurate representation of the structural reliability of dental ceramics [26,27]. The former is a measure of the distribution of flaws, generally for brittle materials, and the latter indicates the 63.21 percentile of the strength distribution [26]. Higher Weibull moduli indicate less variability (greater reliability) between the strength values of specimens in the same group, corresponding to an enhanced structural integrity of the material [6,28].

In a study by Fischer et al. [23], the shear strength of veneering ceramics intended for use with zirconia ranged from 21.9 to 31.0 MPa. In this study, the mean SBS values for all groups tested, except for group EL3, were within this range (Table 2), although the test design was different from that of the study. However, the Weibull moduli were substantially different among the groups tested. It is still uncertain whether the bonding nature between the liner (or porcelain) and zirconia is mechanical or chemical [12], although it is possible that chemical bonding is the major bonding factor [23]. However, the wetting behavior is of primary importance for the good adhesion of veneering porcelain onto zirconia substructure [20]. When the wetting is poor, the application of a liner over the zirconia surface may reduce the porcelain/zirconia interfacial toughness [18]. The wetting behavior is dependent not only on the liner composition but also on the morphology and surface energy of the zirconia material [18]. As shown in Figure 1b, the wetting of CL seems to be relatively poor, probably due to the CL’s high viscosity and different chemical composition from the zirconia material [19]. Thus, it seems that the application of CL onto zirconia prior to veneering impaired the structural integrity between zirconia and veneering porcelain, as expressed by a low Weibull modulus.

On the contrary, glycerol-based ELs seem to have better wettability, probably due to their low viscosity and additionally due to chemical modification in the case of ELs containing zirconia (Figure 1c). In the SBS test, the Weibull modulus of group EL0 was similar to that of group NL (Table 2). This may have been due to the good wetting of the EL, even without zirconia incorporated. Moreover, the enhanced bonding by the EL3 application, expressed as the highest SBS value and Weibull modulus, may be attributed to the optimal mechanical reinforcement by the incorporated zirconia in addition to favorable wetting [10]. However, the Weibull moduli decreased again with an increase in the amount the incorporated zirconia (groups EL6 and EL9).

The CTE for the core material and veneering ceramic should closely match to achieve strong interfacial bond strength [6,10,12]. A high stress area is created and concentrated close to the interface when there is an increased CTE mismatch between the two materials [2], potentially causing a veneering ceramic fracture [6,9,29,30]. The use of a veneering porcelain with a slightly lower CTE than that of the zirconia framework results in a desirable residual compressive stress in the porcelain [10]. In this study, the CTEs of the liners were not found to be critical in determining the zirconia/porcelain bonding behaviors (Table 2, Table 3 and Table 4). It seems that the liner layer allowed a more gradual transition between zirconia and veneering porcelain in terms of CTE. However, small differences in CTE through the porcelain layer and between the porcelain and zirconia may be accentuated because the CTE is not linear with temperature change [16].

In this study, a TPFS test was used to evaluate the strength of a porcelain/zirconia bilayer ceramic system (Figure 3). It has been reported that premature porcelain fractures start from the subsurface of the veneer or from the interface with the core [6,18]. Cracking of the porcelain suggests a high concentration of residual stresses that allow pre-existent flaws to propagate by slow crack growth until failure [6,15]. During three-point bending, the surface of porcelain, where maximum tensile stresses are generated, will tend to fail more easily because porcelain is less resistant to tensile stress than to compressive stress [2,6]. Therefore, the porcelain was tested under tension in the present study. The clinically recommended occlusal thickness for a veneered zirconia restoration is approximately 2 mm [31]. Benetti et al. [6] reported that the mean flexural strengths of a ceramic system decreased as the porcelain veneer thickness increased, but there was no significant strength difference between porcelain thicknesses of 1 and 2 mm. Thus, a zirconia thickness of 0.7 mm, the minimum thickness for appropriate strength at occlusal areas [6], and a porcelain thickness of 1.0 mm was used in this study. It has also been reported that a faster cooling rate increases the temperature gradients and, as a result, residual thermal stress inside the porcelain [15,16]. In this study, the veneering porcelain and liners (including ELs) were fired according to the respective manufactures’ recommendations (Table 5). Cooling is often accelerated to shorten the time required for the fabrication of prosthetic restorations in the laboratory [32]. The control of thermal stress, by the use of slow-cooling protocols and the avoidance of thermal gradients inside the porcelain, has been reported as a success factor in zirconia-based restoration [14]. Such factors (thickness and cooling protocol of veneer ceramic) were not primarily tested in the present study, but they require further investigation.

The TPFS test results (Figure 2 and Table 4), which were generally consistent with those of the SBS test (Table 2), clearly show that the EL3 application significantly improved the flexural strength (91.9 ± 14.0 MPa) of the bilayer ceramic system when compared with the other two experimental conditions (NL: 57.2 ± 9.3 MPa; CL: 52.4 ± 17.6 MPa). However, the fracture behavior of a bilayer ceramic system should not be characterized only by the flexural strength values [26]. Although groups NL and CL showed statistically similar flexural strength values, the former exhibited a higher Weibull modulus (*m* = 6.0) than the latter group (*m* = 2.7). Group EL3 showed the highest Weibull modulus (*m* = 7.3) among the three groups tested. Thus, the Weibull analysis results for the TPFS test were also similar to those for the SBS test (Table 2). A high Weibull modulus (*m* ≥ 20) indicates a small error range and a high level of structural integrity of the material and great reliability [25,26]. The Weibull modulus value lower than 20 for group EL3 suggests that the bilayer system possesses less-than-ideal homogeneity. A recent study suggested that multiple layers and firings increased the flexural resistance when compared to a single firing in a bilayer ceramic specimen [33]. Thus, the formulation, application method, or firing procedure of EL3 still needs some modification or improvement to maximize the reliability of the bilayer ceramic system. 

The fractographic analysis results were also consistent with the flexural strength values (Table 4). For most of the bilayer ceramic specimens, predominant failure mode was cracking. In group EL3, the specimens that failed catastrophically consistently showed higher flexural strength values than the other specimens that showed cracked porcelain. Among the six specimens, no sign of fracture (chipping, cracking, or delamination) was detected before the catastrophic failure and only a partial delamination was detected after the zirconia substructure fracture (Figure 2c). In addition, the fractured interface surfaces of the zirconia substructures were covered with residual veneer and liner layers (Figure 2c). These findings suggest improved structural integrity of the zirconia/porcelain bilayer ceramic system due to the application of EL3 to the porcelain veneering.

It has been suggested that the high moisture content and veneering temperature during the wet veneering procedure facilitate the generation of grain faceting on zirconia surface and, as a result, a decrease in the zirconia/porcelain interfacial bonding [2,6,11]. This phenomenon, related to phase transformation, may be more obvious when using porcelain materials with higher liquid content [11]. Although this aspect was not addressed in the present study, it requires further investigation. In addition, only one commercial zirconia, one commercial veneering ceramic, and one CL were used in this study (Table 1). Various commercial zirconia all-ceramic systems may produce different bond strengths to veneering porcelain [2]. It is also advised that liner material should not be used in combination with pressable veneering porcelain, as this definitely impairs the integration between zirconia and porcelain [2,22]. These results might be attributable to the lower strength of liner materials as compared to dentin ceramics [10]. As the present study did not include various commercial ceramic products for each type, further studies are needed to better confirm the effectiveness of the novel experimental ceramic liner.

## 4. Materials and Methods

### 4.1. Shear Bond Strength (SBS) Test

A total of 90 cubic-shaped (20 mm × 20 mm × 20 mm) zirconia blocks (Cercon ht, Dentsply International Inc., York, PA, USA) were designed in AutoCAD software (Autodesk Inc., Mill Valley, CA, USA), milled using a milling machine (Yenadent D40 series, Yena Makina, Istanbul, Turkey), and sintered in a furnace (LHT 04/16, Nabertherm, Lilienthal, Germany) according to the manufacturer’s instructions. Prior to bonding (veneering), all zirconia specimens were ultrasonically cleaned, air-dried, and randomly divided into six groups, containing 15 specimens each.

**Figure 4:**
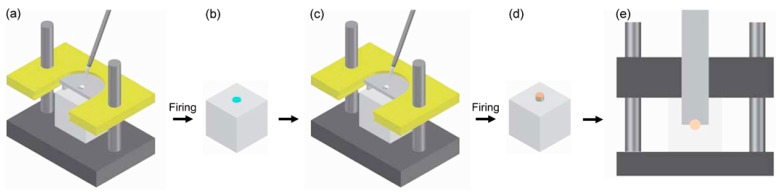
Fabrication process of the shear bond strength test specimen using Ultradent jig. (**a**) Application of a liner on an isolated zirconia surface; (**b**) Liner-coated zirconia specimen; (**c**) Application of veneering porcelain on the liner; (**d**) Veneered zirconia specimen; and (**e**) Shear bond strength testing in a universal testing machine. For group NL (no-liner), the veneering porcelain was directly applied onto the zirconia surface without the liner application and firing steps.

The bonding procedures were performed using an Ultradent jig (Ultradent Products Inc., South Jordan, UT, USA), as shown in Figure 4. The zirconia specimen was placed into the jig with a milled surface facing upward, the surface being isolated using a cylindrical-shaped plastic matrix (diameter: 2.38 mm). In group NL (no-liner), a mixed porcelain slurry (Ceramco PFZ, Ceramco, Burlington, NJ, USA) was directly applied to the surface through the matrix. After excess liquid was removed, the specimen was taken from the jig and then fired in a porcelain furnace (Vacumat 40 T, Vita Zahnfabrik, Bad Säckingen, Germany) according to the manufacturer’s recommendation (Table 5). In the liner application groups, a thin coat of a CL paste (HeraCeram Zr-Adhesive, Heraeus Kulzer GmbH, Hanau, Germany) or one of the ELs was applied onto the zirconia surface through the matrix. The liner-coated zirconia specimen was removed from the jig and then fired in the porcelain furnace according to the recommendation of the manufacturer of CL (Table 5). After the firing of the liner, the specimen was placed again into the jig, a mixed porcelain slurry (Ceramco PFZ) was applied onto the liner region through the matrix, and it then fired as described above.

The specimens were perpendicularly engaged at their veneered ceramic cylinder bases with a round-notched custom shear blade (Ultradent Products Inc., South Jordan, UT, USA) in a universal testing machine (3343, Instron Inc., Canton, MA, USA) at a crosshead speed of 0.5 mm/min until bonding failure occurred (Figure 4) [34]. SBS values in MPa were calculated from the peak load of failure (N) divided by the veneered surface area, the diameter (~2.38 mm) of which for each specimen had been measured using a Vernier caliper prior to debonding. Following debonding, all fractured surfaces were examined under a stereomicroscope (SZ61, Olympus, Tokyo, Japan) at 40× magnification. The failure modes were classified into one of the three types: adhesive failure at the porcelain/zirconia interface; cohesive failure within the porcelain or zirconia; and a combination of these two failure modes (mixed failure).

### 4.2. Coefficient of Linear Thermal Expansion (CTE)

The CTEs of the zirconia, veneering ceramic, CL, and EL3 used in this study were measured using a thermomechanical analyzer (TMA-60H, Shimadzu Corp., Kyoto, Japan). For each material, five rod-shaped specimens (10 mm in length, 5 mm in diameter) were prepared. The ends of the specimens were ground with abrasive papers so that they were flat, parallel and perpendicular to the axis of the specimens. After each specimen was placed in the TMA oven, the length was measured using the software’s “read length” function. An expansion measurement of the test specimen was performed at 10 °C/min between 25 °C and 500 °C. For each specimen, the CTE between 25 °C and 500 °C was determined by referring to plotted curves indicating the expansion in relation to the temperature.

### 4.3. Three-Point Flexural Strength (TPFS) Test

A total of 45 bar-shaped zirconia specimens were prepared in the same way as the SBS test specimens. The dimensions (20 mm × 4.0 mm × 0.7 mm) of each specimen were checked with a Vernier caliper [6]. All zirconia specimens were ultrasonically cleaned, air-dried, and randomly divided into three groups, containing 15 specimens each: one no-liner application group (NL) and two liner application groups (CL and EL3). For the latter two groups, the corresponding liner was applied on the zirconia surface (20 mm × 4.0 mm) and then fired following a protocol similar to the previously described method. A mixed porcelain slurry was applied on the surface of the zirconia specimen, which had been placed into a metal mold for adequate control of the veneering porcelain thickness (1.0 mm) [6]. After excess liquid was removed, the specimen was removed from the mold and fired. The porcelain was applied in two states to compensate for shrinkage after firing [35]. Specimens were polished using silicon carbide paper up to 1200 grit and then 1 μm diamond paste [6]. After the edges of the specimens were rounded [25], the final dimensions were checked with a vernier caliper prior to testing. For each group, two specimens were additionally prepared for the observation of as-milled or liner-applied zirconia surface and cross-sectional morphology of the zirconia/porcelain interface. The specimens were observed by a field emission scanning electron microscope (FE-SEM, JSM-6700F, Jeol, Tokyo, Japan) after sputter-coating with platinum.

The bilayer ceramic specimens were then subjected to a TPFS test, with the porcelain layer under tension [6], using the universal testing machine (Figure 3). The load was applied on the specimen at the midpoint between the support rollers (crosshead speed = 0.5 mm/min). The maximum load in N was recorded at the first sign of fracture, as verified by sound emission and a sudden change in the force-displacement curve [6]. The flexural strength (in MPa) was calculated in accordance with the equations of Benetti et al. [6]. The fractured specimens were examined under the stereomicroscope at a magnification of 40× to determine the failure modes. These were classified as: chipping when it began in the veneering porcelain surface without exposure of the zirconia substructure; cracking when the veneering porcelain cracked at the interface; delamination when the veneering porcelain was damaged and the zirconia substructure was exposed; and catastrophic when fracture occurred in both the veneering porcelain and the zirconia substructure [6,14].

### 4.4. Statistical Analysis

The data (SBS, CTE, and TPFS values) were examined for the normality of distribution with the Shapiro-Wilk test and the equality of variances with the Levene test. The data set, which met both assumptions, was then subjected to a one-way ANOVA followed by Tukey’s post hoc test (α = 0.05) using SPSS 18.0 for Windows software (SPSS Inc., Chicago, IL, USA). The SBS and TPFS results were also analyzed using Weibull distribution to determine the shape parameters (Weibull moduli, *m*) and scale parameters (characteristic strengths, *σ*_0_).

## 5. Conclusions

Within the limitations of this in vitro study, the novel ceramic liner containing 3.0 wt % zirconia significantly enhanced the zirconia/porcelain interfacial bonding. On the contrary, the commercial liner application did not show any positive effect on the bond strength nor flexural strength values. Considering the multifactorial and complex nature of the fracture behavior of veneered zirconia restorations, in vivo studies are still required to better understand the clinical significance of the findings of this in vitro study.

## Figures and Tables

**Table 1:** Ceramic materials used in this study.

Material	Brand Name	Manufacturer	Chemical Composition	Batch Number
Zirconia	Cercon ht	Dentsply International Inc., York, PA, USA	ZrO_2_ (94%), Y_2_O_3_ (5%), Al_2_O_3_ (<1%), Si_2_O_3_ (<1%) ^1^	18016353
Veneering ceramic (porcelain)	Ceramco PFZ	Dentsply Ceramco, Burlington, NJ, USA	SiO_2_, Al_2_O_3_, Na_2_O, K_2_O, SnO_2_, CeO_2_, pigments, 1.3-Butanediol Xi ^1^	12001410
Commercial liner	HeraCeram Zr-Adhesive	Heraeus Kulzer, GmbH, Hanau, Germany	Not disclosed by the manufacturer	2710
Experimental liners	Not applicable	Glycerol: Wako, Tokyo, Japan; ZrO_2_: Tosho Co., Tokyo, Japan; SiO_2_: Wako; Al_2_O_3_ and Na_3_AlF_6_: Junsei, Tokyo, Japan	ZrO_2_, SiO_2_, Al_2_O_3_, Na_3_AlF_6_ (glycerol-based)	Glycerol: PEH4633; ZrO_2_: S303555B; SiO_2_: DG2751; Al_2_O_3_: 2014B1581; Na_3_AlF_6_: 2011G1149

^1^ Provided by the manufacturer.

**Table 2:** Shear bond strength for each group (*n* = 15).

Groups	Shear Bond Strength ^1^	Weibull Distribution Values ^2^				Failure Mode ^3^		
	Mean ± SD in MPa	*m*	*σ*_0_ (MPa)	*σ*_0.05_ (MPa)	*σ*_0.01_ (MPa)	Adhesive	Cohesive	Mixed
NL	23.6 ± 3.5 ^a^	7.6	25.1	17.0	13.7	0	0	15
CL	23.0 ± 5.2 ^a^	4.7	25.2	13.4	9.5	3	0	12
EL0	25.2 ± 3.8 ^a^	7.4	26.8	17.9	14.4	0	0	15
EL3	34.2 ± 4.3 ^b^	8.9	36.1	25.9	21.5	0	0	15
EL6	24.1 ± 4.5 ^a^	5.9	25.9	15.7	11.9	1	0	14
EL9	22.2 ± 3.7 ^a^	6.2	23.2	14.4	11.0	0	0	15

^1^ Values followed by identical lowercase superscripted letters (a and b) indicate no statistical differences (*p* > 0.05). ^2^ *m*: shape parameter (Weibull modulus); *σ*_0_: scale parameter (characteristic strength); *σ*_0.05_ and *σ*_0.01_: probability of failure at 5% and at 1%, respectively. ^3^ Number of specimens.

**Table 3:** Coefficient of linear thermal expansion of the four ceramic materials used (*n* = 5).

Material Type	Brand Name or Code	Mean ± SD in 10^−6^ K^−1^
Zirconia	Cercon ht	10.7 ± 0.1 ^a,^^1^
Veneering porcelain	Ceramco PFZ	9.1 ± 0.1 ^b^
Commercial liner	HeraCeram Zr-Adhesive	9.6 ± 0.1 ^c^
Experimental liner	EL3	9.5 ± 0.2 ^c^

^1^ Values followed by identical lowercase superscripted letters (a, b, and c) indicate no statistical differences (*p* > 0.05).

**Table 4:** Flexural strength, Weibull distribution values, and failure mode distribution for each group (*n* = 15).

Groups	Flexural Strength ^1^	Weibull Distribution Values ^2^				Failure Mode ^3^			
	Mean ± SD in MPa	*m*	*σ*_0_ (MPa)	*σ*_0.05_ (MPa)	*σ*_0.01_ (MPa)	A	B	C	D
NL	57.2 ± 9.3 ^a^	6.0	61.6	37.5	28.6	0	15	0	0
CL	52.4 ± 17.6 ^a^	2.7	60.0	20.0	10.9	0	14	1	0
EL3	91.9 ± 14.0 ^b^	7.3	97.9	65.2	52.1	0	9	0	6

^1^ Values followed by identical lowercase superscripted letters (a and b) indicate no statistical differences (*p* > 0.05). ^2^ *m*: shape parameter (Weibull modulus); *σ*_0_: scale parameter (characteristic strength); *σ*_0.05_ and *σ*_0.01_: probability of failure at 5% and at 1%, respectively. ^3^ Number of specimens. A: Chipping; B: Cracking; C: Delamination; and D: Catastrophic.

**Table 5:** Firing parameters of the veneering porcelain (Ceramco PFZ) and liners (HeraCeram Zr-Adhesive and ELs) used.

Materials	Drying Time (min)	Pre-Heating Time (min)	Starting Temp. (°C)	Heating Rate (°C/min)	Vacuum Start Temp. (°C)	Vacuum Stop Temp. (°C)	Final Temp. (°C)	Holding Time	Cooling Time (min)
Veneering porcelain	5	5	450	60	450	900	900	15 s	0
Liners	5	1	600	100	600	-	1050	10 min	0
